# Humoral Immunogenicity and Reactogenicity of the Standard ChAdOx1 nCoV-19 Vaccination in Taiwan

**DOI:** 10.3390/vaccines10020312

**Published:** 2022-02-17

**Authors:** Jer-Hwa Chang, Jeng-Fong Chiou, Ching-Sheng Hung, Ming-Che Liu, Hui-Wen Chang, Shiao-Ya Hong, Cheng-Yi Wang, Yi-Ling Lin, Yi-Chen Hsieh, Chi-Li Chung, Ying-Shih Su, Shu-Tai Shen Hsiao, Doresses Liu, Jian-Jong Liang, Chun-Che Liao, Chih-Shin Chang, Kevin Shu-Leung Lai, Han-Chuan Chuang, Ko-Ling Chien, Wei-Ciao Wu, Yuan-Chii G. Lee, Sey-En Lin, Yung-Kang Shen, Chiung-Fang Hsu, Jude Chu-Chun Wang, Shih-Hsin Hsiao

**Affiliations:** 1 School of Respiratory Therapy, College of Medicine, Taipei Medical University, Taipei 11031, Taiwan; m102094030@tmu.edu.tw; 2 Division of Pulmonary Medicine, Department of Internal Medicine, Wan Fang Hospital, Taipei Medical University, Taipei 11696, Taiwan; 3 Department of Radiation Oncology, Taipei Medical University Hospital, Taipei 11031, Taiwan; solomanc@tmu.edu.tw; 4 Department of Radiology, School of Medicine, College of Medicine, Taipei Medical University, Taipei 11031, Taiwan; 5 School of Medical Laboratory Science and Biotechnology, College of Medical Science and Technology, Taipei Medical University, Taipei 11031, Taiwan; oryx@w.tmu.edu.tw (C.-S.H.); g160090005@tmu.edu.tw (H.-W.C.); 6 Department of Laboratory Medicine, Wan Fang Hospital, Taipei Medical University, Taipei 11696, Taiwan; 7 Department of Urology, Taipei Medical University Hospital, Taipei 11031, Taiwan; d204097002@tmu.edu.tw; 8 School of Dental Technology, College of Oral Medicine, Taipei Medical University, Taipei 11031, Taiwan; ykshen@tmu.edu.tw; 9 Department of Medical Laboratory, Taipei Medical University Hospital, Taipei 11031, Taiwan; 10 Medical Research Center, Cardinal Tien Hospital, New Taipei 24148, Taiwan; f91445113@ntu.edu.tw; 11 Department of Internal Medicine, Cardinal Tien Hospital, New Taipei 23148, Taiwan; 072782@mail.fju.edu.tw; 12 School of Medicine, College of Medicine, Fu Jen Catholic University, New Taipei 24205, Taiwan; 13 Institute of Biomedical Sciences, Academia Sinica, Taipei 11529, Taiwan; yll@ibms.sinica.edu.tw (Y.-L.L.); jjliang@ibms.sinica.edu.tw (J.-J.L.); jfliao@ibms.sinica.edu.tw (C.-C.L.); 14 Biomedical Translation Research Center, Academia Sinica, Taipei 11529, Taiwan; shin@ibms.sinica.edu.tw; 15 Ph.D. Program of Neural Regenerative Medicine, College of Medical Science and Technology, Taipei Medical University, Taipei 11031, Taiwan; ychsieh@tmu.edu.tw; 16 Master Program in Applied Molecular Epidemiology, College of Public Health, Taipei Medical University, Taipei 11031, Taiwan; 17 Division of Pulmonary Medicine, Department of Internal Medicine, School of Medicine, College of Medicine, Taipei Medical University, Taipei 11031, Taiwan; clchung@tmu.edu.tw; 18 Division of Pulmonary Medicine, Department of Internal Medicine, Taipei Medical University Hospital, Taipei 11031, Taiwan; 983010@tmuh.org.tw (K.S.-L.L.); 153093@h.tmu.edu.tw (K.-L.C.); katharine@tmu.edu.tw (C.-F.H.); 195018@h.tmu.edu.tw (J.C.-C.W.); 19 Division of Infectious Disease, Department of Internal Medicine, Wan Fang Hospital, Taipei Medical University, Taipei 11696, Taiwan; 109005@w.tmu.edu.tw (Y.-S.S.); 151007@h.tmu.edu.tw (H.-C.C.); 20 Department of Internal Medicine, School of Medicine, College of Medicine, Taipei Medical University, Taipei 11031, Taiwan; 21 Department of Nursing, Taipei Medical University Hospital, Taipei 11031, Taiwan; debrasth@h.tmu.edu.tw; 22 School of Nursing, College of Nursing, Taipei Medical University, Taipei 11031, Taiwan; 23 Department of Nursing, Wan Fang Hospital, Taipei Medical University, Taipei 11696, Taiwan; 101043@w.tmu.edu.tw; 24 Center for Nursing and Healthcare Research in Clinical Practice Application, Wan Fang Hospital, Taipei Medical University, Taipei 11696, Taiwan; 25 Department of Critical Care Medicine, Taipei Medical University Hospital, Taipei 11031, Taiwan; 26 Graduate Institute of Medical Sciences, College of Medicine, Taipei Medical University, Taipei 11031, Taiwan; d119107002@tmu.edu.tw; 27 Department of Thoracic Surgery, Department of Surgery, Shuang Ho Hospital, Taipei Medical University, Taipei 23561, Taiwan; 28 Graduate Institute of Biomedical Informatics, College of Medical Science and Technology, Taipei Medical University, Taipei 11031, Taiwan; ycgl@tmu.edu.tw; 29 Department of Anatomic Pathology, New Taipei Municipal TuCheng Hospital, Chang Gung Memorial Foundation, New Taipei 23652, Taiwan; hayasi@tmu.edu.tw

**Keywords:** ChAdOx1 nCoV-19, vaccine, Asian populations

## Abstract

Background: The ChAdOx1 nCoV-19 vaccine has been widely administered against SARS-CoV-2 infection; however, data regarding its immunogenicity, reactogenicity, and potential differences in responses among Asian populations remain scarce. Methods: 270 participants without prior COVID-19 were enrolled to receive ChAdOx1 nCoV-19 vaccination with a prime–boost interval of 8–9 weeks. Their specific SARS-CoV-2 antibodies, neutralizing antibody titers (NT50), platelet counts, and D-dimer levels were analyzed before and after vaccination. Results: The seroconversion rates of anti-RBD and anti-spike IgG at day 28 after a boost vaccination (BD28) were 100% and 95.19%, respectively. Anti-RBD and anti-spike IgG levels were highly correlated (r = 0.7891), which were 172.9 ± 170.4 and 179.3 ± 76.88 BAU/mL at BD28, respectively. The geometric mean concentrations (GMCs) of NT50 for all participants increased to 132.9 IU/mL (95% CI 120.0–147.1) at BD28 and were highly correlated with anti-RBD and anti-spike IgG levels (r = 0.8248 and 0.7474, respectively). Body weight index was statistically significantly associated with anti-RBD IgG levels (*p* = 0.035), while female recipients had higher anti-spike IgG levels (*p* = 0.038). The GMCs of NT50 declined with age (*p* = 0.0163) and were significantly different across age groups (159.7 IU/mL for 20–29 years, 99.4 IU/mL for ≥50 years, *p* = 0.0026). Injection-site pain, fever, and fatigue were the major reactogenicity, which were more pronounced after prime vaccination and in younger participants (<50 years). Platelet counts decreased and D-dimer levels increased after vaccination but were not clinically relevant. No serious adverse events or deaths were observed. Conclusion: The vaccine is well-tolerated and elicited robust humoral immunity against SARS-CoV-2 after standard prime–boost vaccination in Taiwanese recipients.

## 1. Introduction

Over a dozen severe acute respiratory syndrome coronavirus 2 (SARS-CoV-2) vaccines have received emergency use authorization (EUA) to combat the COVID-19 pandemic [1]. To expedite the development of SARS-CoV-2 vaccines during the heyday of the COVID-19 pandemic in 2020, most clinical trials were conducted in limited countries [1]. Consequently, some vaccines lack sufficient data on diverse ethnicities even after the EUA [2].

A diverse mix of ethnicities is mandatory to meaningfully assess the vaccine efficacy (VE) and safety in phase 3 clinical trial to facilitate subgroup analysis for any potential difference among different ethnic groups [2]. A slight difference in VE between ethnic groups has been observed in the BNT162 mRNA COVID-19 vaccine trial [3]. The ChAdOx1 nCoV-19 vaccine has received EUA from 121 governments worldwide, including in Asia where nearly 60% of the global population lives. Due to the urgency of the pandemic, Asian governments granted EUA to the ChAdOx1 nCoV-19 vaccine based on the results of clinical trials conducted in non-Asian countries [4,5], where Asians accounted for only 4.4% of participants [2]. To better achieve global immunity, further investigation of safety, efficacy, and immunogenicity of the ChAdOx1 nCoV-19 vaccine in Asian populations is required to identify whether there is a need to tailor vaccine programs in Asian populations.

SARS-CoV-2 enters human cells through the fusion of its receptor-binding domain (RBD) of surface spike glycoprotein and host cellular angiotensin converting enzyme 2 (ACE2) receptors [6]; it can then be eradicated by innate and adaptive immune responses [7]. Induction of humoral immunity against SARS-CoV-2 through natural infection or vaccination has been shown to reduce the risk of reinfection and COVID-19 symptoms [8]. It has been known that many variables, including intrinsic host and extrinsic factors, vaccine contents and administration, may influence the immune response to a vaccine [8]. However, whether individual characteristics of vaccine recipients, including age, body mass index (BMI), and chronic comorbidities (CCM), influence the immunogenicity of the ChAdOx1 nCoV-19 vaccine remains mostly unknown for Asian populations. In this prospective study, we investigated the immunogenicity and reactogenicity profiles of vaccine recipients in Taiwan after the standard prime–boost ChAdOx1 nCoV-19 vaccination.

## 2. Study Designs and Methods

### 2.1. Ethical Approval and Study Subjects

This prospective study was conducted in 2 tertiary hospitals following the principles of the Declaration of Helsinki and Good Clinical Practice under the approval of Taipei Medical University Joint Institutional Review Board (TMU-VIM-001). Eligible participants were adults who were aged 20–65 years (amended to 20–85 years later) and not pregnant, scheduled to receive a prime–boost ChAdOx1 nCoV-19 vaccination regimen, and had no severe active diseases (e.g., advanced-stage malignancy, profound immune disorders), prior history of COVID-19 diagnosis or SARS-CoV-2 vaccine administration. Volunteers with new onset of fever, cough, shortness of breath, or anosmia within 14 days before enrollment were excluded. The status of prior COVID-19 disease and SARS-CoV-2 vaccination was determined by medical history review and serum anti-RBD IgG level test. Administration of any other SARS-CoV-2 vaccine was prohibited, but sexual activity and medicine were not limited during the study period. Between 22 March and 2 April 2021, 272 eligible participants were enrolled. They were all healthcare workers or contract employees working at the hospital. Everyone received a prime ChAdOx1 nCoV-19 vaccination. Two participants did not have a boost vaccination and were withdrawn; one became pregnant 1 month after her prime vaccination and the other was afraid of the reactogenicity induced by a boost vaccination.

### 2.2. Study Design

Every volunteer underwent a screening visit, where inclusion/exclusion criteria and medical history were assessed. All participants provided written informed consent and were not misinformed. All eligible participants were scheduled to receive the prime–boost ChAdOx1 nCoV-19 vaccination at the standard dose of 0.5 mL. Vaccines were administered into the deltoid as a single intramuscular injection. All recipients stayed at the clinics for 15–30 min to monitor immediate reactions after vaccine administration. The interval between the prime and boost doses (PB interval) was 8 to 9 weeks according to the ChAdOx1 nCoV-19 vaccine manufacturer guidelines and government policy. All participants had blood drawn 0–7 days before the prime dose of ChAdOx1 nCoV-19 vaccine (PD0) and 28 days after the boost dose (BD28). The blood draw and tests 14 days after the prime were optional (PD14). Participants had clinical assessments for safety at days 0, 14, 56, or 63 after prime vaccination and at day 28 after the booster. The solicited reactogenicity of the prime–boost ChAdOx1 nCoV-19 vaccination was self-reported through questionnaire (potential sources of bias). Blood clots and relevant medical events after vaccination were determined through blood tests and electronic medical record review. Whether participants were infected during the study period (defined as from PD0 to BD28) was determined by testing serum specific anti-SARS-CoV-2-nucleocapsid (N) protein (negative result defined as cut-off index <1.0 according to the manufacturer’s protocol).

### 2.3. Serological Assays

Serological in vitro diagnostic (IVD) assays for anti-RBD and anti-N protein IgG was performed using the SARS-CoV-2 IgG II assay (Abbott, Sligo, Ireland) and Elecsys anti-SARS-CoV-2 N protein immunoassay (Roche Diagnostics, Basel, Switzerland), respectively. The anti-RBD cut off of ≥50 AU/mL, i.e., 7 BAU/mL (AU/mL × 0.142 can be converted to BAU/mL), and the anti-N protein cut-off of ≥1 U/mL were defined as a positive result according to the manufacturers’ guidelines. The subclass antibody responses against SARS-CoV-2 (anti-RBD, anti-spike IgG, IgA, IgM) were detected by in-house enzyme-linked immunosorbent assay (ELISA). Receiver operating curve (ROC) analysis was used to determine the best cut-off value for distinguishing vaccine-induced humoral immunity from baseline with high sensitivity and specificity. NT50 (50% neutralizing titer) was determined as the highest dilution titer that could inhibit 50% of the cytopathic effect in the SARS-CoV-2 live virus neutralization assay. The antibody concentrations and neutralizing antibody activity against the original SARS-CoV-2 were presented in international units, BAU/mL and IU/mL, respectively.

### 2.4. Enzyme-Linked Immunosorbent Assay

The 96-well microplates (Thermo Fisher Scientific, Waltham, MA, USA) were coated with 50 μL of 1 μg/mL SARS-CoV-2 specific spike RBD or spike (10500-CV, 10549-CV, R&D Systems, Minneapolis, MN, USA) in PBS and 50 μL 2× coating buffer (Cat. No. 421701, Biolegend, San Diego, CA, USA) and left overnight at 4 °C. The next day, the plates were washed twice with PBST (0.1% Tween-20 in PBS) and incubated with blocking solution (2% BSA in PBS) for 1 h. After rinsing three times with PBST, 100 μL serum dilutions (final dilution 1:1000 in blocking solution) were added and incubated at RT for 1 h. The plates were washed five times, followed by incubation with 100 μL of 50 ng/mL biotin-labeled goat anti-human Ig (109-066-170, 109-066-129 and 109-066-011, Jackson ImmunoResearch, West Grove, PA, USA) at RT for 1 h. The plates were washed five times and 100 µL of 1 µg/mL avidin-horseradish peroxidase (016-030-084, Jackson ImmunoResearch) was added at RT for 30 min. The plates were washed five times and 50 μL of TMB substrate (Cat. No. 421101, BioLegend) was added. After incubation at RT for 5–10 min (IgG/IgM 5 min, IgA 10 min), 50 μL of 0.2 N H_2_SO_4_ was added to stop the reaction. The optical density (OD) of SARS-CoV-2 specific IgG, IgA, and IgM antibodies was measured at 450 nm with a microplate reader (Biotek Synergy, Winooski, VT, USA). The standard curve was prepared by making 2-fold serial dilutions (from 1:100 to 1:12,800) of the WHO recommended National Institute for Biological Standards and Control (NIBSC) 20/136. The OD values of the samples were then converted into binding antibody units (BAU)/ml by comparison with the OD values of the standard using four-parameter nonlinear regression (GraphPad Prism v.8). The inter-assay %CV was <15% while the intra-assay %CV was less than 10% to ensure the overall reliability of the immunoassay results.

### 2.5. SARS-CoV-2 Live Virus Neutralization Assay

The neutralizing antibody titer was determined using a microneutralization test based on cytopathic effect. Wild-type SARS-CoV-2 (TCDC#4) was amplified and titrated on Vero-E6 cells (ATCC CRL-1586) to obtain the viral titer (TCID50/mL). One day before the neutralization assay, Vero-E6 cells (1.2 × 10^4^ cells/well) were cultured in DMEM (Hyclone) supplemented with 10% fetal bovine serum (FBS, Gibco, Carlsbad, CA, USA) and 1× Penicillin-Streptomycin solution (Thermo Fisher Scientific) in a humidified incubator with 5% CO_2_ at 37 °C overnight. Tested sera were heated at 56 °C for 30 min to inactivate complement and four-fold diluted in DMEM supplemented with 2% FBS and 1× Penicillin/Streptomycin. The sera then underwent two-fold serial dilution up to 2048-fold of dilution. Fifty microliters of diluted sera were mixed with an equal volume of virus (100 TCID50 in DMEM with 2% FBS) and incubated at 37 °C for 1 h. Final sera dilutions in the sera–virus mixture ranged from 1:8 to 1:4096. After removing the overnight culture medium, 100 μL of the sera–virus mixtures were inoculated onto a confluent monolayer of Vero-E6 cells in quadruplicate. After incubation for 4 days, the cells were fixed with 10% formaldehyde and stained with 0.5% crystal violet staining solution at room temperature for 20 min. Individual wells were then scored for CPE as having a binary outcome of ‘infection’ or ‘no infection’ and the neutralizing antibody titers were calculated according to Reed and Muench. The reciprocal of the highest dilution capable of inhibiting 50% of the cytopathic effect was defined as 50% neutralizing titer (NT50). A panel of the NIBSC standards 20/268, 20/130, and 20/136, were analyzed by using the same validated assays and used to convert the data of NT50 to IU/mL.

### 2.6. Statistics and Analyses

Baseline descriptive characteristics of the study population were expressed as frequency with percentages for categorical data and mean with standard deviation for continuous data. The assessment of the change in immunogenicity between two vaccinations was analyzed using paired *t*-test. An independent student’s *t*-test was used to examine the immunogenicity between two groups; analysis of variance (ANOVA) was used to test 3 or 4 groups. Spearman’s correlation coefficient was performed to estimate the correlation between immunogenicity levels. The model was adjusted for age, sex, BMI, and presence of CCM. The ROC and the 95% confidence interval (CI) for the mean of antibody was analyzed using GraphPad Prism 8.0.1 (GraphPad, San Diego, CA, USA). All other statistical analyses were performed using SAS Version 9.4 (SAS Institute, Cary, NC, USA) considering two-sided probabilities with a *p* value less than 0.05. 

## 3. Results

A total of 270 participants (mean age 38.72 ± 12.22 years, range 23–68 years) completed the standard ChAdOx1 nCoV-19 vaccination regimen with a mean PB interval of 61.83 ± 2.85 days (Table 1) and were assessable for immunogenicity profile at PD0 and BD28. Among them, 243 participants could be assessed at PD14. Each blood sample collected at BD28 (*n* = 270) was negative for the presence of a specific anti-N protein IgG against SARS-CoV-2, indicating that no participant was infected by SARS-CoV-2 during the study period.

**Figure 1:**
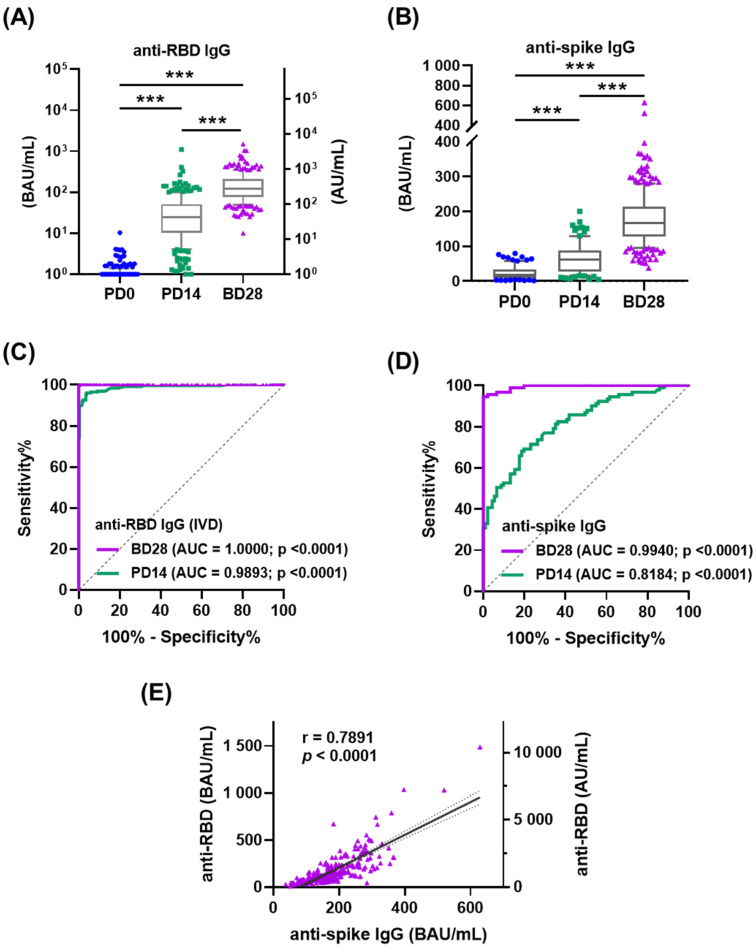
Antibody responses in ChAdOx1 nCoV-19 vaccinated recipients without previous COVID-19. Participant serum samples were collected at PD0, PD14, and BD28. PD0: day 0 before the prime vaccination; PD14: 14 days after the prime vaccination; BD28: 28 days after the boost vaccination. (**A**,**B**) Anti-spike RBD (**C**) and anti-spike (**D**) IgG levels were measured before and after vaccination. AU: arbitrary units; BAU: binding antibody units. (**C**,**D**) Receiver operating curves (ROC) for anti-RBD (**A**) and anti-spike (**B**) IgG were analyzed to assess the predicted seropositivity after vaccination. (**E**) Linear regression analysis was performed to show the correlation of anti-spike RBD and anti-spike IgG. *** *p* < 0.001.

All participants elicited high levels of anti-RBD and anti-spike IgG at PD14 (45.02 ± 85.32 and 64.40 ± 42.96 BAU/mL), and much higher levels at BD28 (172.87 ± 170.36 and 179.30 ± 76.88 BAU/mL) (Figure 1A,B and Tables S1 and S2). The sensitivity and specificity of anti-RBD and anti-spike IgG antibodies were determined by antibody levels in participant serum at PD0, PD14, and BD28 to maximize seropositivity. ROC analysis demonstrated the AUC between PD0 and BD28 was better than that between PD0 and PD14, with 1.0000 for anti-RBD IgG and 0.9940 for anti-spike IgG (Figure 1C,D). The sensitivity and specificity of Abbott RBD IgG detection (cut off ≥ 7 BAU/mL) were 99.37% and 99.55%, respectively, according to the manufacturer’s data. We used a cut-off value of 78.31 BAU/mL for anti-spike IgG, achieving a sensitivity of 95% and a specificity of 99%. Each baseline serum sample was negative for anti-RBD and anti-spike IgG (0.85 ± 0.88 and 23.67 ± 19.74 BAU/mL) (Figure 1C,D and Tables S1 and S2), reinforcing participant self-reports of no prior COVID-19 and not having previously joined SARS-CoV-2 vaccine trials. There was a strong positive correlation between anti-RBD and anti-spike IgG levels induced by the prime–boost ChAdOx1 nCoV-19 vaccination regimen (r = 0.7891; *p* < 0.001) (Figure 1E).

The seroconversion rates of anti-RBD IgG were 82.79% (202/244) at PD14 and 100% (270/270) at DB28; proportions of seroconversion anti-spike IgG samples were 32.97% (30/91) at PD14 and 95.19% (257/270) at DB28 (data not shown). Subgroup analysis showed that BMI had statistical association with differences in anti-RBD IgG levels at BD28 (*p* = 0.035); gender, age, and CCM history were not statistically associated (Table 2). Conversely, gender was statistically associated with differences in anti-spike IgG levels at BD28 (*p* = 0.038); age, BMI, CCM history were not (Table 2). Individual IgM and IgA responses were also measured randomly by ELISA in one-third of participants to examine the composition of the total serological response (Figure S1). Serum anti-RBD IgM and IgA at PD0 were 77.43 ± 59.81 and 72.20 ± 66.08 BAU/mL, and anti-spike IgM and IgA were 66.46 ± 78.98 and 62.33 ± 67.25 BAU/mL. The slightly higher concentrations of anti-RBD and anti-spike spike IgM and IgA were observed at PD14 followed by a decrease at DB28 (Figure S1).

**Figure 2:**
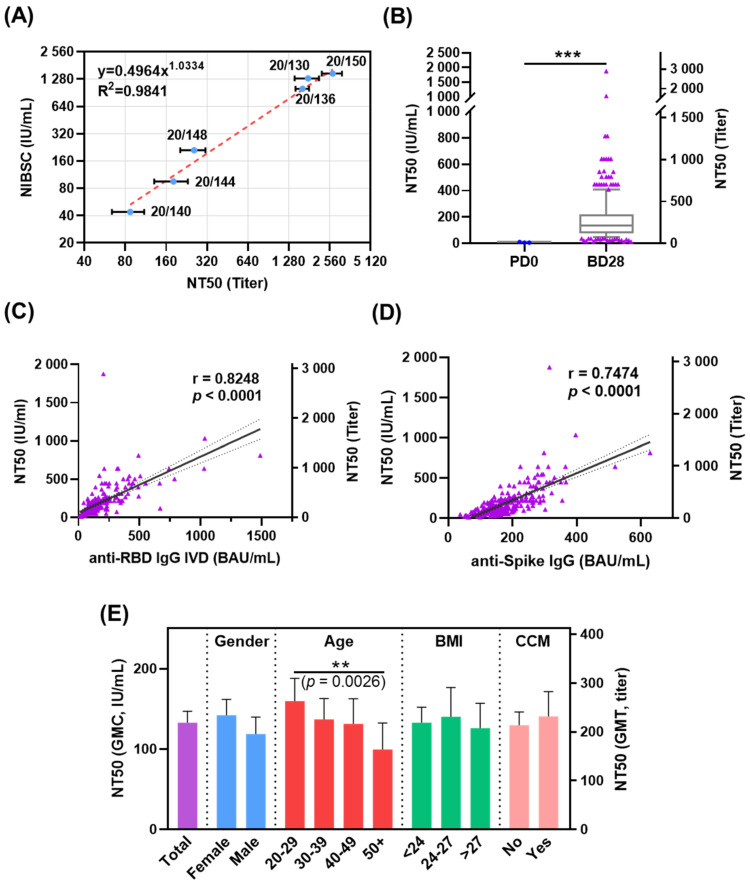
Neutralizing activity of vaccine-elicited antibody. (**A**) Neutralizing antibody titer (NT50) of NIBSC standards was measured by SARS-CoV-2 live virus neutralization assay. (**B**) NT50 of serum samples collected from participants at PD0 and BD28. (**C**,**D**) Linear regression analyses showed the correlation of NT50 against anti-spike RBD (**C**) or anti-spike IgG (**D**). (**E**) The GMCs or GMTs and 95% CI of NT50 against SARS-CoV-2 in subgroups. ** *p* < 0.01; *** *p* < 0.001.

A live SARS-CoV-2 microneutralization assay was conducted to assess the neutralizing antibody titers of vaccine recipients. The NT50 titers for the NIBSC standards were determined to define the conversion of NT50 titers to IU/mL (linear regression R^2^ = 0.9841) (Figure 2A). Three of the 270 participants showed slightly higher NT50 levels of 6.09, 6.09, and 10.28 IU/mL (<4.25 IU/mL was identified as a negative result), respectively, at PD0 with unknown etiology (Figure 2B), but their anti-RBD IgG and anti-spike IgG levels at PD0, and anti-N IgG at BD28 were negative. Following two doses of vaccination, the geometric mean concentrations (GMCs) of NT50 in all participants increased to 132.88 IU/mL (95% CI 120.0–147.1) at DB28 (Figure 2B and Table S3). NT50 was highly correlated with anti-RBD (r = 0.8248, *p* < 0.0001, Figure 2C) or anti-spike IgG levels (r = 0.7474, *p* < 0.0001, Figure 2D), respectively. Subgroup analyses showed that the GMCs declined with age (*p* for trend = 0.0163) and were significantly different among age subgroups (Figure 2E, Table S3). Younger participants (20–29 years) displayed a higher GMC of NT50 than those ≥50 years [159.7 IU/mL (95% CI 135.9–187.8) and 99.4 IU/mL (95% CI 74.6–132.5), *p* = 0.0026, Figure 2E and Table S3]. The same results were found when the neutralizing antibody activities were presented with geometric mean titer (GMT) (Figure 2E, Table S3). Use of antipyretics before or after vaccination showed no association with vaccine-elicited neutralizing antibody responses (data not shown).

The solicited reactogenicity after a prime–boost vaccination, not including the severity of local and systemic adverse effects (AEs), was surveyed through a questionnaire and reported by 248 (91.9%) participants (Table 3). Pain at the injection site was the most frequent local AE (77.82% after the prime dose and 59.68% after the boost dose), and swelling at the injection site was commonly reported after the prime and boost doses (43.39% and 27.32%, respectively). Younger age was associated with a higher frequency of local AEs than those over 50 after the prime dose (85.71% vs. 62.0%, *p* = 0.0001) and after the boost dose (67.51% vs. 47.6%, *p* = 0.007). CCM were not associated with local AE occurrence (data not shown). Fatigue, general weakness, fever, chills, and headache were the most common systemic AEs of the prime–boost ChAdOx1 nCoV-19 vaccination regimen reported. Fatigue was reported in 196 (79.03%) participants after the prime dose and 122 (49.19%) after the boost dose. A report of fever (defined as ≥37.5 °C in the questionnaire) was also common after the prime dose but less common after the boost dose (54.44 vs. 18.55%). Body temperature ≥38.5 °C was reported in 32 (12.96%) participants after a prime dose and in 3 (1.21%) participants after a boost dose. Five participants visited the emergency department for fever within 24 h after the prime vaccination; all of them felt much better after taking antipyretics and left the emergency department quickly. There were a few reports of chest pain, abdominal pain, and dyspnea. The frequency of antipyretics use before and after vaccinations was also reported by participants. Nearly half (47.98%) of participants took antipyretics after the prime dose and a third (32.93%) after the boost dose. One hundred and seven participants reported headache after the prime vaccination and one visited OPD due to severe headache by day 4 after prime vaccination. Both the laboratory tests and the brain scan imaging study revealed no specific abnormality and her headache improved after taking antipyretics. Compared with younger participants, older participants were associated with a lower frequency of systemic AEs, especially fever, fatigue, and chills (*p* < 0.0001). CCM were not associated with systemic AE occurrence (data not shown). Overall, the reactogenicity after the boost vaccination was milder than that after the prime vaccination and no serious AEs were reported.

**Figure 3:**
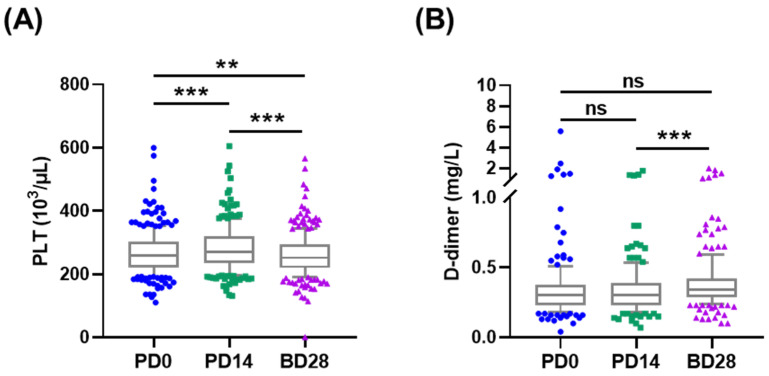
Platelet counts and D-dimer responses induced by vaccination. (**A**) Platelet counts and (**B**) D-dimer levels were tested at PD0, PD14, and BD28. ** *p* < 0.01; *** *p* < 0.001.

Platelet counts and D-dimer levels were also tested for vaccine-induced thrombotic thrombocytopenia. Platelet counts increased from (267.24 ± 67.91) × 10^3^/uL at PD0 to (281.80 ± 71.78) × 10^3^/uL at PD14, then decreased to (261.18 ± 67.05) × 10^3^/uL at BD28, all within the normal range 130–400 × 10^3^/uL (Figure 3A and Table S4). Subgroup analyses showed similar results. The D-dimer level at BD28 (0.36 ± 0.21 mg/L) was higher than at PD14 (0.32 ± 0.18 mg/L) and PD0 (0.34 ± 0.39 mg/L) (*p* < 0.0001, Table S5). Subgroup analyses showed similar results. No thromboembolic events were reported by participants or by medical record review.

## 4. Discussion

Previous studies have shown that prior SARS-CoV-2 infection [9] and the PB interval of the ChAdOx1 nCoV-19 vaccination [5] significantly influenced the humoral immune responses to the vaccine, which may lead to biases in evaluating the immunogenicity of vaccine recipients. Our prospective study reported the humoral immunogenicity and reactogenicity profiles of 270 participants who had no prior COVID-19, received a standard two ChAdOx1 nCoV-19 doses regimen with a strict PB interval (mean 61.83 ± 2.86 days), and were not infected by SARS-CoV-2 during the study period in Taiwan. Given the extremely low prevalence of SARS-CoV-2 infection in Taiwan (62.9 per 100,000 residents on 25 June 2021), our cohort represented a pristine, virus-naïve population. Additionally, the serological immune response data were presented in units suggested by the WHO for convenient comparison with independent studies worldwide.

The most common local and systemic reactogenicity profiles of the ChAdOx1 nCoV-19 vaccine observed in our cohorts included injection-site pain, fatigue, fever, and headache, which were similar to those in the UK cohort [4]. The frequency of reactogenicity after the boost vaccination or in the older adults (≥50 years) were lower, consistent with previous publications on ChAdOx1 nCoV-19 vaccination [4]. In contrast, mRNA-based boost SARS-CoV-2 vaccinations caused more severe and frequent systemic reactogenicity than the prime dose [3,10]. Vaccine-induced immune thrombotic thrombocytopenia (VITT) is a rare but life-threatening AE, and it was reported to be one case per 100,000 ChAdOx1 nCoV-19 vaccination [11]. The median platelet count at diagnosis is approximately 20,000 to 30,000 per cubic millimeter (range, approximately 10,000 to 110,000) [11]. Decreased platelet counts and increased D-dimer levels were observed after vaccination in our cohort (Tables S5 and S6); however, the differences were small, and no clinical significance was found.

Whether ethnicity impacts the vaccine response has been an area of interest. The standard prime–boost ChAdOx1 nCoV-19 vaccination was immunogenic and further elicited a significant boost effect on anti-RBD and anti-spike IgG responses at BD28, in line with that in the non-Asian dominant study cohort [4]. The seroconversion rate of anti-spike and anti-RBD IgG reached to 95.19% and 100% at BD28 in our cohort, indicating that the ChAdOx1 nCoV-19 vaccine could evoke good immunogenicity not only in the Caucasian population but also in the Taiwanese population, and further supporting the EUA of the ChAdOx1 nCoV-19 vaccine in Taiwan. Since the anti-RBD IgG levels in the 50+ age group might be lower than that in other age groups at PD14, the anti-RBD IgG levels in the 50+ age group was similar as that in other age groups at BD28, indicating that the boost vaccination is especially necessary in the 50+ age group. The role of IgM response has also been studied in a small series of 20 volunteers receiving mRNA-based vaccination [12]. In our cohort, anti-RBD IgM and IgA, and anti-spike IgM and IgA did not demonstrate significant increase after the boost dose, suggesting that they might not be a good indicator for humoral immunogenicity induced by the standard ChAdOx1 nCoV-19 vaccination. The neutralizing activity of the ChAdOx1 nCoV-19 vaccination at BD28 of our study cohort (*n* = 270) as measured by the SARS-CoV-2 live virus neutralization assay was 100%, consistent with that (>99%) of the UK study cohort (*n* = 209) [4]. This further supported that ChAdOx1 nCoV-19 vaccination can evoke general humoral immunogenicity across distinct cohorts. A pooled analyses of four randomized ChAdOx1 nCoV-19 vaccine trials conducted in non-Asian countries showed that the overall VE against symptomatic COVID-19 was 81.3% (95% CI 60.3–91.2) in participants administered two vaccine doses with an ≥12-week PB interval, and was 55.1% (95% CI 33.0–69.9) in those with an <6-week PB interval [13]. Nonetheless, in the UK and Brazil phase 2/3 trials, only 517 of the 11,636 participants (4.4%) enrolled to receive two doses of ChAdOx1 nCoV-19 vaccines/placebo were of Asian origins, and the VE for this minor group was not independently reported [4,5]. Recently, neutralizing antibody titers, anti-RBD, and anti-spike IgG evoked by COVID-19 vaccines were reported to be highly correlated with immune protection against symptomatic SARS-CoV-2 infection [14,15]. The analyses of the ChAdOx1 nCoV-19 phase 3 trial demonstrated that the GMT of NT50 at 135–247, anti-RBD IgG at 165–506 BAU/mL, and anti-spike IgG at 113–264 BAU/mL corresponded to 70–80% VE, respectively (20). Applying the same concept, the overall estimated VE of the standard prime–boost ChAdOx1 nCoV-19 vaccination against the original SARS-CoV-2 in our cohort may be around 70–80%, which was in line with the results of non-Asian dominant phase 2/3 trials [5]. The speculation above may further support the EUA of the standard ChAdOx1 nCoV-19 vaccination in Taiwan.

Previous reports have shown that the humoral immunity evoked by ChAdOx1 nCoV-19 vaccines varied greatly among individuals [4,16]. Individual characteristics, such as age, gender, and BMI were reported to be related to the susceptibility and severity of COVID-19 [17,18,19]. Older adults are more likely to be infected by SARS-CoV-2 compared to young adults [17]. A phase 2/3 ChAdOx1 nCoV-19 vaccine trial showed that normalized neutralizing titers of 126 non-Asian participants were similar across age groups (*p* = 0.40) [4]. On the other hand, a phase 1 trial of 45 healthy participants who received the prime–boost mRNA1273 vaccination demonstrated similar immunogenicity between different age groups [20], but one real-world data indicated that the immunogenicity induced by one dose of BNT162b2 mRNA COVID-19 vaccine decreased with age [9]. An observational study has also shown that age and gender, but not BMI, are associated with statistically significant differences in antibody response after BNT162b2 vaccination [21]. Our subgroup analyses revealed a statistically significant association between BMI and anti-RBD IgG levels, while gender was statistically associated with the difference in anti-spike IgG levels. The GMC of NT50 declined with age (trend *p* = 0.0163) and was significantly different across age groups (e.g., 159.7 IU/mL for 20–29 years and 99.4 IU/mL for ≥50 years, *p* = 0.0158). In addition, although the anti-RBD IgG level was associated with BMI in our cohort, the prime–boost ChAdOx1 nCoV-19 vaccination could successfully evoke similar neutralizing antibody activities against SARS-CoV-2 (Figure 2E), regardless of participants’ BMIs. Moreover, female recipients in our cohort had stronger specific antibody responses to the ChAdOx1 nCoV-19 vaccine at DB28 in terms of anti-spike IgG levels but not anti-RBD IgG levels or NT50 (Table 2 and Table S3), similar to the BNT COVID-19 vaccine eliciting higher anti-spike IgG levels at BD28 in women than men [22]. Furthermore, participants with fever (body temperature ≥37.5 °C) after vaccination were associated with higher NT50 (*p* = 0.0196, Table S6), which may be confounded by age because younger recipients in our cohort were more likely to have a fever. Nevertheless, the impact of individual characteristics on the vaccine response remains inconclusive. Further exploration may be needed while assessing who needs a second boost dose (third vaccination).

The limitations of our study include, but are not limited to, the scarcity of older participants (>65 years), the lack of antibody neutralizing activity data before the boost vaccination, and cellular immunity response profiles. In addition, the study design did not allow for distinguishing VE for SARS-CoV-2 variants. A longer follow-up could provide additional information regarding the presumed duration of vaccine-acquired immunity.

## 5. Conclusions

The reactogenicity and humoral immunogenicity profiles of ChAdOx1 nCoV-19 vaccine recipients in this cohort provided useful information for local authorities and other Asian governments to better understand the potential risks and beneficial effects of the ChAdOx1 nCoV-19 vaccine on Asian populations.

## Figures and Tables

**Table 1:** Baseline characteristics of vaccination recipients.

Variable	Total *n* = 270	
Age mean (SD), years	38.65	(12.14)
Age group, *n* (%)		
20–30	79	(29.26)
30–39	75	(27.78)
40–49	61	(22.59)
50+	55	(20.37)
Gender, *n* (%)		
Female	169	(62.59)
Male	101	(37.41)
BMI mean (SD), kg/m^2^	23.87	(4.17)
BMI group, *n* (%)		
<24	153	(56.67)
24–27	58	(21.48)
>27	59	(21.85)
Duration between 2 vaccines		
Mean (SD), days	61.83	(2.86)
Median (IQR), days	63	(61–64)
Hematological parameters, mean (SD)		
WBC, 10^3^/uL	6.58	(1.71)
HGB, g/dL	13.68	(1.57)
PLT, ×10^3^/uL	267.24	(67.91)
D-dimer, mg/L	0.31	(0.40)
CCM history ^#^, *n* (%)		
No	190	(70.37)
Yes	80	(29.63)
Anti-RBD IgG before enrollment (Prior COVID-19 ≥ 7), mean (SD), BAU/mL	0.85	(0.8)
Anti-N IgM/G during study ^##^ (SARS-CoV-2 infection ≥ 1.0), mean (SD), U/mL	0.10	(0.03)

^#^ Asthma, hypertension, diabetes, hyperlipidemia, hepatitis B, hepatitis C, hypertensive heart disease, colon cancer, thyroid cancer, Sjogren’s syndrome, morbid obesity; ^##^ The test result of recipients’ blood collected at 28 days after the boost vaccination.

**Table 2:** Subgroup analyses of anti-RBD and anti-spike IgG levels after the prime–boost vaccination.

	*n*	Anti-RBD IgG (IVD)			Anti-Spike IgG		
		Mean (SD)		*p*-Value	Mean (SD)		*p*-Value
All participants	270	172.87	(170.36)		179.30	(76.88)	
Gender				0.306			0.038
Female	169	181.08	(179.18)		186.79	(77.98)	
Male	101	159.12	(154.38)		166.76	(73.69)	
Age, years				0.793			0.972
20–29	79	174.48	(117.23)		182.82	(66.92)	
30–39	75	156.69	(156.84)		177.74	(63.52)	
40–49	61	183.41	(184.36)		178.09	(84.96)	
50+	55	180.92	(229.56)		177.69	(96.96)	
BMI, kg/m^2^				0.035			0.056
<24	153	158.70	(137.60)		170.77	(66.92)	
24–27	58	224.11	(262.49)		198.89	(97.01)	
>27	59	159.25	(118.02)		182.14	(76.46)	
CCM history				0.053			0.059
No	190	156.67	(156.76)		173.62	(75.63)	
Yes	80	203.11	(189.04)		192.79	(78.62)	

Note: The levels of anti-RBD and anti-spike IgG presented the immune responses at 28 days after the boost vaccination. BMI: body mass index; CCM: chronic comorbidities; IVD: in vitro diagnostic; SD: standard deviation. (Unit: BAU/mL).

**Table 3:** Solicited reactogenicity of the prime–boost vaccinations and antipyretics use between participants aged <50 years and ≥50 years.

Variable	Prime							Boost						
	Total		<50		≥50		*p*-Value	Total		<50		≥50		*p*-Value
	n	(%)	n	(%)	n	(%)		n	(%)	n	(%)	n	(%)	
Injection site	199	(80.89)	168	(85.71)	31	(62.00)	0.0001	157	(63.31)	133	(67.51)	24	(47.06)	0.007
Pain	193	(77.82)	164	(82.83)	29	(58.00)	0.0002	148	(59.68)	126	(63.96)	22	(43.14)	0.007
Redness	81	(32.79)	70	(35.53)	11	(22.00)	0.069	47	(18.95)	38	(19.29)	9	(17.65)	0.790
Swelling	122	(49.39)	110	(55.84)	12	(24.00)	<0.0001	69	(27.82)	60	(30.46)	9	(17.65)	0.069
Fever	135	(54.44)	123	(62.44)	12	(24.00)	<0.0001	46	(18.55)	41	(20.81)	5	(9.80)	0.071
37.5–38 °C	58	(23.48)	49	(24.87)	9	(18.00)	0.306	31	(12.50)	26	(13.20)	5	(9.80)	0.514
38.1–38.5 °C	46	(18.55)	45	(22.84)	1	(2.00)	0.001	12	(4.84)	12	(6.09)	0	(0.00)	0.134
>38.5 °C	32	(12.96)	30	(15.23)	2	(4.00)	0.035	3	(1.21)	3	(1.52)	0	(0.00)	1.000
Fatigue	196	(79.03)	171	(86.36)	25	(50.00)	<0.0001	122	(49.19)	108	(54.82)	14	(27.45)	0.001
General weakness	153	(61.69)	137	(69.19)	16	(32.00)	<0.0001	64	(25.81)	57	(28.93)	7	(13.73)	0.027
Chills	129	(52.02)	119	(60.10)	10	(20.00)	<0.0001	51	(20.56)	47	(23.86)	4	(7.84)	0.012
Headache	107	(43.32)	97	(49.24)	10	(20.00)	0.0002	60	(24.19)	54	(27.41)	6	(11.76)	0.020
Chest pain	8	(3.23)	7	(3.54)	1	(2.00)	1.000	8	(3.23)	7	(3.55)	1	(1.96)	1.000
Abdominal pain	7	(2.82)	7	(100.00)	0	(0.00)	0.350	4	(1.61)	4	(2.03)	0	(0.00)	0.584
Dyspnea	8	(3.23)	7	(3.54)	1	(2.00)	1.000	3	(1.21)	3	(1.52)	0	(0.00)	1.000
Antipyretics use before vaccination	18	(7.26)	16	(8.08)	2	(4.00)	0.541	35	(14.23)	32	(16.33)	3	(6.00)	0.062
Antipyretics use after vaccination	119	(47.98)	110	(55.56)	9	(18.00)	<0.0001	81	(32.93)	76	(38.78)	5	(10.00)	0.0001

## Data Availability

The original data presented in the study are included in the article, further inquiries can be directed to the corresponding author.

## Supplementary Materials

The following supporting information can be downloaded at: https://www.mdpi.com/article/10.3390/vaccines10020312/s1, Table S1. Anti-RBD IgG (IVD) levels at different time points before and after vaccination. Table S2. Anti-spike IgG levels at different time points before and after vaccination. Table S3. Subgroup analyses of NT50 levels after the prime-boost vaccination. Table S4. Platelet counts at different time points before and after vaccination. Table S5. D-dimer levels at differ time points before and after vaccination. Table S6. The association of antibody responses, platelet counts and D-dimer levels with fever after vaccination. Figure S1. Subclass IgM and IgA responses elicited by vaccination. IgA and IgM levels of anti-Spike RBD (A,B) and anti-spike (C,D) were measured randomly by ELISA in one-third of participants (91/270). PD0: day 0 before the prime vaccination; PD14: 14 days after the prime vaccination; BD28: 28 days after the boost vaccination.
