# Supplementary material for: Humoral Immunogenicity and Reactogenicity of the Standard ChAdOx1 nCoV-19 Vaccination in Taiwan
Source: Vaccines (Basel). 2022 Feb 17;10(2):312. doi: 10.3390/vaccines10020312 (PMC8874464; doi:10.3390/vaccines10020312)
Supplement: Supplementary file 1 [file vaccines-10-00312-s001.zip › vaccines-1579961-supplementary.pdf]

Humoral immunogenicity and reactogenicity of the standard ChAdOx1 nCoV-19 vaccination in Taiwan, an Asian island with extremely low COVID prevalence

Table S1. Anti-RBD IgG (IVD) levels at different time points before and after vaccination

	**PD0**		**PD14**		**BD28**		***p*-value[a]**	***p*-value[b]**	***p*-value[c]**
	n	**Mean (SD)**	***n***	**Mean (SD)**	***n***	**Mean (SD)**			
All participants	270	0.85 (0.88)	243	45.02 (85.32)	270	172.87 (170.36)	<0.0001	<0.0001	<0.0001
Gender									
Female	169	0.91 (1.00)	153	45.78 (57.20)	169	181.08 (179.18)	<0.0001	<0.0001	<0.0001
Male	101	0.77 (0.61)	90	43.75 (119.18)	101	159.12 (154.38)	0.0010	<0.0001	<0.0001
Age, year									
20–29	79	0.75 (0.59)	66	52.95 (57.91)	79	174.48 (117.23)	<0.0001	<0.0001	<0.0001
30–39	75	0.83 (0.64)	69	40.31 (53.07)	75	156.69 (156.84)	<0.0001	<0.0001	<0.0001
40–49	61	0.89 (1.33)	57	60.80 (149.73)	61	183.41 (184.36)	0.0038	<0.0001	<0.0001
50+	55	1.02 (0.87)	51	23.51 (32.66)	55	180.92 (229.56)	<0.0001	<0.0001	<0.0001
BMI, kg/m^2									
<24	153	0.93 (1.05)	135	40.86 (48.44)	153	158.70 (137.60)	<0.0001	<0.0001	<0.0001
24–27	58	0.86 (0.71)	53	61.14 (152.96)	58	224.11 (262.49)	0.0059	<0.0001	<0.0001
>27	59	0.68 (0.38)	55	39.71 (62.79)	59	159.25 (118.02)	<0.0001	<0.0001	<0.0001
CCM history									
No	190	0.88 (0.94)	171	39.52 (49.70)	189	159.39 (159.29)	<0.0001	<0.0001	<0.0001
Yes	80	0.83 (0.71)	72	58.10 (136.57)	80	206.02 (191.74)	0.0007	<0.0001	<0.0001

Note: PD0: day 0 before the prime vaccination; PD14: 14 days after the prime vaccination; DB28: 28 days after the boost vaccination. BMI: body mass index; CCM: chronic comorbidities; SD: standard deviation. The positive immune response is ≥7 BAU/mL. [a]PD0 v.s. PD14; [b]PD14 v.s. BD28; [c]PD0 v.s. BD28. (Unit: BAU/mL)

Table S2. Anti-spike IgG levels at different time points before and after vaccination

	PD0		PD14		BD28		*p*-value[a]	*p*-value[b]	*p*-value[c]
	n	**Mean (SD)**	***n***	**Mean (SD)**	***n***	**Mean (SD)**			
All participants	91	23.67 (19.74)	91	64.40 (42.96)	270	179.30 (76.88)	<0.0001	<0.0001	<0.0001
Gender									
Female	56	25.23 (21.22)	56	64.27 (42.36)	169	186.79 (77.98)	<0.0001	<0.0001	<0.0001
Male	35	21.18 (17.10)	35	64.59 (44.52)	101	166.76 (73.69)	<0.0001	<0.0001	<0.0001
Age, years									
20–29	26	25.83 (20.82)	26	73.53 (38.29)	79	182.82 (66.92)	<0.0001	<0.0001	<0.0001
30–39	24	24.45 (19.87)	24	72.20 (52.38)	75	177.74 (63.52)	<0.0001	<0.0001	<0.0001
40–49	19	29.87 (22.77)	19	65.46 (42.11)	61	178.09 (84.96)	0.0005	<0.0001	<0.0001
50+	22	14.93 (12.45)	22	44.17 (32.17)	55	177.69 (96.96)	<0.0001	<0.0001	<0.0001
BMI, kg/m^2									
<24	47	21.18 (18.51)	47	61.11 (44.29)	153	170.77 (66.92)	<0.0001	<0.0001	<0.0001
24–27	25	22.72 (18.32)	25	74.23 (42.41)	58	198.89 (97.01)	<0.0001	<0.0001	<0.0001
>27	19	31.11 (23.41)	19	59.59 (40.36)	59	182.14 (76.46)	0.0045	<0.0001	<0.0001
CCM history									
No	65	19.76 (17.34)	65	61.18 (43.71)	190	173.62 (75.63)	<0.0001	<0.0001	<0.0001
Yes	26	33.45 (22.20)	26	72.43 (40.71)	80	192.79 (78.62)	<0.0001	<0.0001	<0.0001

Note: PD0: day 0 before the prime vaccination; PD14: 14 days after the prime vaccination; DB28: 28 days after the boost vaccination. Tests were conducted randomly in one-third of blood specimens collected at PD0 and PD14 (91/270). BMI: body mass index; CCM: chronic comorbidities; SD: standard deviation. The positive immune response is ≥78.31 BAU/mL. [a]PD0 v.s. PD14; [b]PD14 v.s. BD28; [c]PD0 v.s. BD28. (Unit: BAU/mL)

Table S3. Subgroup analyses of NT50 levels after the prime-boost vaccination

	n	Mean (SD)		*p*-value	GM (95% CI)		*p*-value
		Titer	IU/mL		Titer	IU/mL	
All participants	270	308.76 (291.23)	187.7 (185.2)		223.4 (202.5−246.5)	132.9 (120.0−147.1)	
Gender				0.069			0.089
Female	169	331.66 (323.26)	202.1 (206.4)		238.7 (210.6−270.5)	142.2 (125.0−161.9)	
Male	101	270.44 (224.13)	163.4 (140.6)		200.1 (170.6−234.8)	118.6 (100.5−139.8)	
Age, years				0.435			0.0163
20–29	79	351.22 (362.75)	214.4 (233.6)		267.0 (228.3−312.2)	159.7 (135.9−187.8)	
30–39	75	300.81 (255.74)	182.4 (161.6)		229.8 (194.0−272.1)	136.8 (114.8−162.9)	
40–49	61	296.78 (228.85)	179.8 (143.2)		220.9 (179.5−271.7)	131.3 (106.0−162.7)	
50+	55	271.89 (283.46)	165.1 (178.4)		168.7 (127.8−222.8)	99.4 (74.58−132.5)	
BMI, kg/m^2				0.685			0.793
<24	153	314.44 (330.26)	191.5 (211.2)		223.4 (196.0−254.7)	132.8 (116.0−152.1)	
24–27	58	322.65 (262.68)	196.1 (165.3)		235.5 (188.8−293.8)	140.3 (111.6−176.3)	
>27	59	280.38 (196.51)	169.4 (122.3)		212.3 (171.7−262.5)	126.1 (101.2−156.9)	
CCM history				0.516			0.477
No	190	302.39 (303.13)	183.2 (193.1)		218.3 (194.6−245.0)	129.8 (115.2−146.1)	
Yes	80	326.06 (262.29)	198.3 (165.6)		236.1 (194.9−285.9)	140.6 (115.4−171.5)	

Note: NT50: 50% Neutralizing antibody titer or concentration (IU/mL) was defined as the reciprocal of the highest dilution capable of inhibiting 50% of cytopathic effect. BMI: body mass index; CCM: chronic comorbidities; SD: standard deviation; GM: geometric mean; CI: confidence interval.

Table S4. Platelet counts at different time points before and after vaccination

	PD0		PD14		BD28		*p*-value[a]	*p*-value[b]	*p*-value[c]
	n	**Mean (SD)**	***n***	**Mean (SD)**	***n***	**Mean (SD)**			
All participants	270	267.24 (67.91)	243	281.80 (71.78)	270	261.98 (65.19)	0.0093	0.0011	0.3587
Gender									
Female	169	283.42 (71.50)	153	299.13 (74.85)	169	276.02 (66.94)	0.0550	0.0037	0.3268
Male	101	240.18 (51.35)	90	252.33 (55.20)	101	238.49 (54.89)	0.1166	0.0843	0.8216
Age, years									
20–29	79	267.98 (56.00)	66	285.80 (61.96)	79	259.83 (49.25)	0.0712	0.0056	0.3330
30–39	75	274.03 (72.70)	69	292.83 (79.50)	75	271.27 (73.66)	0.1404	0.0933	0.8177
40–49	61	277.95 (73.54)	57	291.40 (73.10)	61	271.34 (69.21)	0.3216	0.1283	0.6097
50+	55	245.05 (67.07)	51	250.97 (64.04)	55	242.02 (65.41)	0.6436	0.4788	0.8109
BMI, kg/m^2									
<24	153	262.48 (58.28)	135	278.02 (65.17)	153	254.01 (55.17)	0.0335	0.0008	0.1924
24–27	58	267.25 (63.69)	53	284.38 (67.47)	58	269.35 (63.12)	0.1717	0.2277	0.8592
>27	59	279.58 (91.04)	55	288.58 (89.87)	59	275.42 (86.09)	0.5967	0.4262	0.7990
CCM history									
No	190	269.73 (64.40)	171	285.06 (68.91)	190	263.14 (60.94)	0.0296	0.0015	0.3057
Yes	80	261.33 (75.72)	72	274.07 (78.13)	80	259.23 (74.68)	0.3093	0.2335	0.8605

Note: PD0: day 0 before the prime vaccination; PD14: 14 days after the prime vaccination; BD28: 28 days after the boost vaccination; BMI: body mass index; CCM: chronic comorbidities; SD: standard deviation. [a]PD0 v.s. PD14; [b]PD14 v.s. BD28; [c]PD0 v.s. BD28. (Unit: 10^3/uL)

Table S5. D-dimer levels at differ time points before and after vaccination

	PD0		PD14		BD28		*p*-value[a]	*p*-value[b]	*p*-value[c]
	n	**Mean (SD)**	***n***	**Mean (SD)**	***n***	**Mean (SD)**			
All participants	270	0.34 (0.39)	242	0.32 (0.18)	270	0.36 (0.21)	0.5190	0.0161	0.3573
Gender									
Female	169	0.34 (0.45)	153	0.33 (0.20)	169	0.37 (0.19)	0.6466	0.0588	0.5444
Male	101	0.33 (0.26)	89	0.31 (0.16)	101	0.35 (0.25)	0.5890	0.1360	0.4303
Age, yr									
20–29	79	0.32 (0.29)	65	0.32 (0.18)	79	0.37 (0.26)	0.9516	0.1703	0.2570
30–39	75	0.38 (0.63)	69	0.32 (0.16)	75	0.33 (0.12)	0.4046	0.5350	0.4944
40–49	61	0.31 (0.10)	57	0.30 (0.09)	61	0.34 (0.11)	0.5735	0.0269	0.0797
50+	55	0.33 (0.27)	51	0.35 (0.27)	55	0.41 (0.30)	0.7569	0.2386	0.1355
BMI, kg/m^2									
<24	153	0.36 (0.50)	134	0.32 (0.22)	153	0.37 (0.25)	0.3963	0.0985	0.8894
24–27	58	0.31 (0.13)	53	0.31 (0.12)	58	0.32 (0.13)	0.9941	0.0367	0.5213
>27	59	0.30 (0.12)	55	0.32 (0.13)	59	0.38 (0.17)	0.3119	0.5179	0.0026
CCM history									
No	190	0.35 (0.44)	170	0.32 (0.17)	190	0.35 (0.20)	0.3861	0.1085	0.9846
Yes	80	0.31 (0.21)	72	0.32 (0.20)	80	0.39 (0.24)	0.6554	0.0629	0.0208

Note: PD0: day 0 before the prime vaccination; PD14: 14 days after the prime vaccination; BD28: 28 days after the boost vaccination. BMI: body mass index; CCM: chronic comorbidities; SD: standard deviation. [a]PD0 v.s. PD14; [b]PD14 v.s. BD28; [c]PD0 v.s. BD28. (Unit: mg/L)

Table S6. The association of antibody responses, platelet counts and D-dimer levels with fever after vaccination.

Variable	With Fever (n=141) Mean (SD)	Without Fever (n=102) Mean (SD)	*p*-value
anti-RBD IgG (BAU/mL)	175.67 (147.02)	168.39 (204.20)	0.7467
anti-spike IgG (BAU/mL)	186.09 (75.28)	170.89 (81.45)	0.0671
NT50 (IU/mL)	215.20 (212.74)	157.45 (150.38)	0.0196
Platelet ($\times 10^3$/uL)	265.79 (64.28)	259.67 (66.92)	0.472
D-dimer (mg/L)	0.35 (0.17)	0.38 (0.28)	0.292

Note: The participant with fever was defined as who had an episode of body temperature ≥37.5°C after the prime or boost or both doses of vaccine. RBD: receptor binding domain; BAU: binding affinity unit; NT: neutralizing antibody titer; IU: international units; SD: standard deviation.

Figure S1

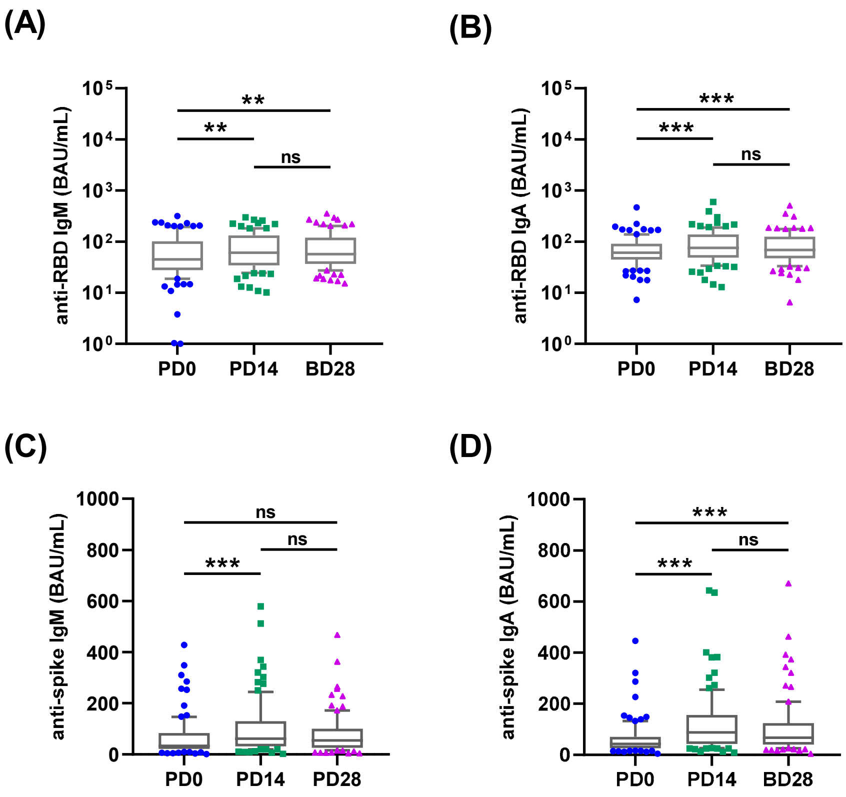

Figure S1. Subclass IgM and IgA responses elicited by vaccination. IgA and IgM levels of anti-Spike RBD (A-B) and anti-spike (C-D) were measured randomly by ELISA in one-third of participants (91/270). PD0: day 0 before the prime vaccination; PD14: 14 days after the prime vaccination; BD28: 28 days after the boost vaccination.
